# Expansion of the human mitochondrial proteome by intra- and inter-compartmental protein duplication

**DOI:** 10.1186/gb-2009-10-11-r135

**Published:** 2009-11-24

**Authors:** Radek Szklarczyk, Martijn A Huynen

**Affiliations:** 1 Centre for Molecular and Biomolecular Informatics, NCMLS, Radboud University Medical Centre, 6500 HB Nijmegen, The Netherlands

## Abstract

**Background:**

Mitochondria are highly complex, membrane-enclosed organelles that are essential to the eukaryotic cell. The experimental elucidation of organellar proteomes combined with the sequencing of complete genomes allows us to trace the evolution of the mitochondrial proteome.

**Results:**

We present a systematic analysis of the evolution of mitochondria via gene duplication in the human lineage. The most common duplications are intra-mitochondrial, in which the ancestral gene and the daughter genes encode mitochondrial proteins. These duplications significantly expanded carbohydrate metabolism, the protein import machinery and the calcium regulation of mitochondrial activity. The second most prevalent duplication, inter-compartmental, extended the catalytic as well as the RNA processing repertoire by the novel mitochondrial localization of the protein encoded by one of the daughter genes. Evaluation of the phylogenetic distribution of N-terminal targeting signals suggests a prompt gain of the novel localization after inter-compartmental duplication. Relocalized duplicates are more often expressed in a tissue-specific manner relative to intra-mitochondrial duplicates and mitochondrial proteins in general. In a number of cases, inter-compartmental duplications can be observed in parallel in yeast and human lineages leading to the convergent evolution of subcellular compartments.

**Conclusions:**

One-to-one human-yeast orthologs are typically restricted to their ancestral subcellular localization. Gene duplication relaxes this constraint on the cellular location, allowing nascent proteins to be relocalized to other compartments. We estimate that the mitochondrial proteome expanded at least 50% since the common ancestor of human and yeast.

## Abstract 2

The human mitochondrial proteome is shown to have expanded due to duplication of protein encoding genes and re-localization of these duplicated proteins.

## Background

Mitochondria, next to their widely recognized function in respiration and ATP production, also play a role in key cellular processes such as lipid metabolism, synthesis of steroid hormones, regulation of apoptosis [1] and calcium signaling [2]. Instrumental to mitochondrial function is the proteome of the organelle, consisting of an estimated 1,500 proteins in human [3]. Recently, owing to advanced proteomics techniques, major progress has been made in elucidating the content of the mammalian mitochondrial proteome. The integration of many types of experimental data and computational predictions resulted in a list of mitochondrial proteins approaching saturation, with a reasonably small false discovery rate of 10% [4]. At the same time analyses of the list of proteins revealed that only a minor fraction of the present day mitochondrial proteome, less than 20%, shows convincing evidence of having originated from the alpha-proteobacterial ancestor [5-7]. This brings the origin of the large majority of mitochondrial proteins into question and suggests that other cellular compartments may have been a source for new mitochondrial proteins. We can examine this hypothesis by comparing organellar proteomes between species.

Detailed, large-scale studies of the inter-species evolution of subcellular localization have begun only recently and have shown conservation between *Schizosaccharomyces pombe *and *Saccharomyces cerevisiae *[8]. There are a number of specific discoveries that indicate that present-day localizations for mitochondrial enzymes and complete pathways do not necessarily reflect their evolutionary origin and there is evidence for the relocalization of multiple metabolic pathways between subcellular compartments. For example, a citrate synthase has been relocalized from mitochondria to the peroxisome in *S. cerevisiae *[9], and most of the proteins that were derived from the ancestor of the mitochondria are not mitochondrial in present day species [6]. It has been observed that Frataxin and Isu1P, which are involved in the iron-sulfur cluster assembly in mitochondria, are localized mainly in the cytosol of the microsporidian species *Trachipleistophora hominis *[10]. After the whole genome duplication event in the ancestor of *S. cerevisiae *a great majority of duplicated genes were purged from the genome [11]. Of those retained, at least 25% functionally diversified via a localization change, altering their amino acid composition, interaction partners and level of expression [12]. But what are the quantitative trends in the evolution of mitochondria in the lineage leading to human?

The composition of the human and mammalian mitochondrial proteome has received great attention in the past years [13-17]. Most recently, probabilistic integrative strategies, which are less plagued with false discoveries specific to any single approach, have allowed the estimation of the mammalian mitochondrial proteome at a level nearing saturation [4]. Next to the human mitochondrion, a wealth of data is available specifically on the localization of mitochondrial proteins in various species: *S. cerevisiae *[18,19], *Arabidopsis thaliana *[20] and *Tetrahymena thermophila *[21]. More than 500 proteins have been found in the mitochondria of the ciliate *T. thermophila *and the estimate for yeast reaches approximately 1,000 proteins [19]. The mammalian mitochondrion is larger still and leads to the question: which biological processes and molecular functions of proteins were introduced to the organelle? Furthermore, how and when were these integrated? We examine the evolutionary history of gene families that contain mitochondrial proteins to answer these questions.

The phylogenomic evidence indicates that the mitochondrial proteome expanded not only by duplications of mitochondrial proteins, but also by relocalizations of paralogs to the organelle, when a copy of a non-mitochondrial protein became targeted to the mitochondrion. We also found that the dates of the appearance of mitochondrial targeting signals indicate that the relocalization of proteins followed promptly after gene duplication.

## Results

Human nuclear-encoded mitochondrial proteins were collected from MitoCarta, the state-of-the-art compendium for the mammalian mitochondrial proteome, created using a combination of experimental identification, bioinformatics analysis, and literature curation [4]. The mitochondrial proteome of *S. cerevisiae*, containing published experimental data [18,22-24] was obtained from the MitoP2 database [25] together with the most comprehensive yeast mitochondrial proteome dataset to date [19]. For the dataset of non-mitochondrial proteins required for our analysis, we used proteins known to localize to 1 of 24 other subcellular compartments (see Materials and methods for details).

### Conservation of mitochondrial localization among one-to-one orthologs

We first ask to what extent mitochondrial localization is conserved between man and yeast for unambiguous one-to-one orthologs that have not been duplicated since the common ancestor of the two species. Mitochondrial localization appears to be very well conserved, with a few notable exceptions. From 143 one-to-one orthologous pairs localized to mitochondria in either of the two species, we find that 124 proteins (87%) are found in this organelle in both species and only 19 proteins localize to mitochondria in one species, but not the other (13%; Table S1 in Additional data file 1). Of the 19 differentially localized proteins, 17 are localized to mitochondria in human and not in yeast, with experimental evidence supporting the localization for all but one protein (Table S1 in Additional data file 1). The two remaining yeast proteins (SEN2 and DNM1), unlike the 17 human mitochondrial proteins, do not enter the yeast mitochondrion, but instead attach to the outer membrane [26,27]. We can infer the ancestral localization of the human mitochondrial proteins by using the *A. thaliana *mitochondrial proteome. Of all 143 unambiguous human-yeast orthologs, 27 proteins were found in plant mitochondria in a liquid chromatography-tandem mass spectrometry study [20], a number that includes only 1 of the 19 differentially localized proteins. With this lack of corroborated mitochondrial localization in the outgroup species, we propose that a gain of mitochondrial localization in the human lineage, rather than a loss in the yeast lineage, has been the main cause of this disparate localization.

A search for a discernible functional coherence among the retargeted proteins revealed the relocalization of a multi-protein functional module in human. Three enzymes participating in ornithine metabolism can be found in mitochondria in human and ureotelic mammals, but not in yeast: OTC, CPSase I and P5CS. Of these, OTC and CPSase I are part of the urea cycle whose evolutionary relocalization has been reported extensively [28,29].

At least 8 of the 17 proteins relocalized in human were concomitantly found in other subcellular compartments of the mammalian cell as indicated in the published literature based on small-scale experiments (Table S2 in Additional data file 1). It should therefore be noted that complete relocalizations to the mitochondria that also involve the loss of the ancestral localization are even more rare than proteins that gain mitochondrial localization without the loss of the ancestral one. Apparently, a protein tends to gain a novel localization without losing the ancestral subcellular localization - for example, by adding a mitochondrial targeting signal to one of its isoforms, as in the case of dUTP pyrophosphatase (DUT) and peroxiredoxin-5 [30,31]. Although interesting in themselves, these observations emphasize that relocalizations of products of single copy genes between subcellular compartments are rare and limited to a relatively small set of cellular functions.

### Increase of the human mitochondrial proteome via intra-mitochondrial protein duplication

**Figure 1:**
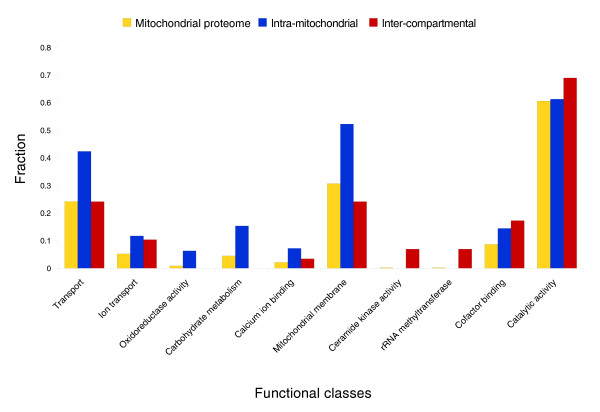
Contribution of different duplication types to the function of the mitochondria. Classes significantly overrepresented compared to the mitochondrial proteome are shown. The height of a bar represents the fraction of proteins that is annotated with a specific category. Three datasets are shown: the whole mitochondrial proteome (MitoCarta proteins [4]; yellow), intra-mitochondrial (blue) and inter-compartmental (red) duplications. Protein functional classes are defined by GO functional classification [68]. Benjamini and Hochberg false discovery rate correction was used to derive statistically significant categories. See Tables S5 and S7 in Additional data file 1 for the full list.

Investigations of the subcellular localization of one-to-one orthologs do not explain the expansion of the mitochondrial proteome. We therefore examined the evolutionary history of duplicated genes containing mitochondrial paralogs. We analyzed eukaryotic gene trees reconciled with the species phylogeny to identify gene duplications that followed the divergence of human and yeast (see Materials and methods for details). We observed two prevailing ways in which gene duplications contributed to the expansion of the metazoan mitochondrial proteome (Table 1). In the first mode, 65 duplications of nuclear genes encoding mitochondrial proteins gave rise to a set of 118 mitochondrial proteins, with up to four proteins per family as in the case of pyruvate dehydrogenases or ADP/ATP translocases (see Table S3 in Additional data file 1 for the list of proteins). With all human paralogs and the yeast ortholog localized to mitochondria, the ancestral protein was most likely targeted to this organelle as well, which is confirmed by the presence of approximately 50% orthologous proteins in plant mitochondria in the study [20]. Figure 1 shows the specific cellular functions performed by intra-mitochondrial protein duplications. A Gene Ontology (GO) analysis reveals enrichment of proteins involved in carbohydrate metabolism ([GO:5975], *P *< 2e-4) and various components of transport ([GO:6810], *P *< 6e-4, amino acid transport, ion transport and protein transport complexes embedded in the inner and outer membranes). Additionally, 11 out of 23 calcium ion binding proteins [GO:5509] originate from intra-mitochondrial duplications (*P *< 7e-4; see Table S5 in Additional data file 1 for the list of all categories). These functional gene classes are significantly overrepresented relative to the composition of the whole mitochondrial proteome, and therefore reflect a specific characteristic of intra-mitochondrial duplications.

**Table 1:** Duplications in gene families with products localized to the mitochondria

Human localization of gene family	Yeast localization of gene family	Number of families	Number of human proteins
Mitochondrial	Mitochondrial	53	118
Mitochondrial and non-mitochondrial	Non-mitochondrial	26	101
Other	Other	25	55

'Mitochondrial' denotes mitochondrial localization for all genes from this family in a species; 'non-mitochondrial' indicates a known localization to another subcellular compartment; 'mitochondrial and non-mitochondrial' indicates families with both mitochondrial and non-mitochondrial paralogs. See also Table S4 in Additional data file 1 for other duplication classes.

### Increase of the human mitochondrial proteome via inter-compartmental protein duplication

The second most common type of duplication associated with increasing the mitochondrial proteome is characterized by human mitochondrial proteins with a human non-mitochondrial paralog (Table 1; Table S6 in Additional data file 1). For those gene families that have a non-mitochondrial ortholog in yeast, the most parsimonious scenario suggests a non-mitochondrial localization in the common ancestor of human and yeast, and a subsequent gain of mitochondrial localization. We hypothesized that these proteins can constitute gains of mitochondrial localization in the human lineage. To validate this hypothesis, we inspected the localization of plant orthologs of inter-compartmental duplications, identifying only two mitochondrial proteins among 29 orthologs in *A. thaliana*. This suggests that the majority of mitochondrial proteins with a non-mitochondrial paralog were ancestrally non-mitochondrial and represent gains of mitochondrial localization in the lineage leading to human. A detailed GO analysis of the entire set of inter-compartmental duplications reveals enrichment among biological processes responsible for molecular functions, such as cofactor binding (*P *< 2e-3, [GO:48037]), intramolecular oxidoreductase (*P *< 5e-3, [GO:16863]), ceramide kinase (*P *< 4e-4, [GO:1729]), catalytic activity in general (*P *< 2e-3, [GO:3824]), but also the process of 12S rRNA methylation (*P *< 4e-3, [GO:154]; Table S7 in Additional data file 1) necessary for the stability of the small ribosomal subunit [32].

The assumption that we can use the non-mitochondrial localization in yeast as a proxy for the ancestral localization enables us to recognize protein retargeting events between mitochondria and other subcellular compartments, including the nucleus (8 out of 29 proteins; Table S8 in Additional data file 1), peroxisome (6 out of 29) and endoplasmic reticulum (5 out of 29 proteins). Four of the six peroxisomal relocalization events encode proteins responsible for fatty acid beta-oxidation in yeast (PCD1, ECI1, DCI1, POX1) and their duplicated orthologs are found in human mitochondria.

### Relocalized proteins often originate from ancient, pre-metazoan duplications

**Figure 2:**
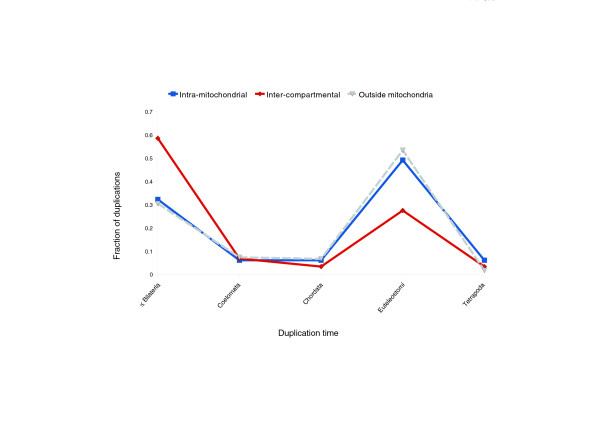
Timing of gene duplications of mitochondrial proteins. The solid blue line represents duplicating mitochondrial proteins, while the solid red line corresponds to duplications of genes followed by relocalization of one of the proteins to the mitochondria. The dashed line denotes protein duplications in other cellular compartments, outside the mitochondria (all proteins are listed in Table S9 in Additional data file 1).

Using phylogenetic trees of genes that encode the modern human mitochondrion, we inferred the timing of duplications (see Materials and methods). Around 80% of duplications are equally divided between two evolutionary stages: before the divergence of bilateria and before the divergence of vertebrates (Figure 2). Intra-mitochondrial gene duplications were found to be representative of the general duplication trends across the whole genome (no statistical difference with the genome-wide duplication trend, *P *> 0.6 Fisher exact test). By contrast, the duplications associated with relocalizations to the mitochondria happened predominantly in the earlier stage of evolution, before the divergence of bilateria. At this evolutionary time point they significantly exceed the genome-wide fraction of duplications (*P *< 0.003). Following the massive duplication events before the radiation of vertebrates (the 2R hypothesis [33,34]; although alternative hypotheses exist [35]), mitochondrial protein content continued to evolve as exemplified by the recent duplication of glutamate dehydrogenase [36]. And even though the reference mitochondrial proteome used in this study is derived from mouse tissues, and therefore the accurate protein localization data for primate-specific duplications is limited, we encountered 16 gene duplications of mitochondrial proteins in primates (Table S11 in Additional data file 1).

### Relocalizations promptly follow duplications

An unmentioned assumption in the analysis of inter-compartmental protein duplications is that the protein relocalization followed shortly after the gene duplication. Even though the pre-sequence mitochondrial import pathway is only one of four presently recognized means of protein import (reviewed in [37]), many mitochondrial proteins contain a short, amino-terminal localization sequence that is indicative of this pathway. This sequence feature is amenable to computational methods [38]. For proteins imported to the mitochondria via the pre-sequence pathway, the gain of a novel localization may be caused by the acquisition of an amino-terminal targeting signal. Indeed, when examining all proteins with a novel mitochondrial localization, a potential mitochondrial targeting signal can be identified in 50% of the proteins, five times more often than in their non-mitochondrial human paralogs (*P *< 0.00005, Fisher exact test). Assuming that in these proteins the targeting signal is responsible for the mitochondrial localization, we examined whether its appearance in evolution coincides with the gene duplication, and thus whether the duplication was concomitant with a gain of mitochondrial localization.

Among human mitochondrial proteins with a non-mitochondrial paralog we find 12 proteins with a recognizable short, amino-terminal targeting sequence. Despite the limitations of computational targeting sequence prediction (for example, [20]) in 9 out of the 12 gene families the phylogenetic analysis indicates that the mitochondrial targeting signal was gained in the same era as, or shortly after, the gene duplication (Table 2).

**Table 2:** Dating of gene duplication of mitochondrial proteins compared to time when the mitochondrial targeting signal appeared in the protein sequence

Paralogs	Duplication before the divergence of	Targeting signal found in
***TOP1MT***, *TOP1*	Vertebrates	Vertebrates (*Gallus gallus*)
***TFB2M***, *DIMT1L*	Animals	Animals (*Anopheles gambiae*)
***NUDT8***, *NUDT7*	Animals	Animals (*Drosophila melanogaster*)
***SIRT3***, *SIRT2*	Coelomata to chordata*	Vertebrates (*Danio rerio*)
***HTRA2***, *HTRA1*	Vertebrates	Vertebrates (*Danio rerio*)
***PDE12***, *CNOT6*	Animals	Chordates (*Ciona intestinalis*)
***PECI***, *CDYL*	Animals	Animals (*Caenorhabditis elegans*)
***HINT2***, *HINT1*	Animals	Animals (*Drosophila melanogaster*)
***GOT2***, *GOT1*	Animals	Animals (*Drosophila melanogaster*)

The names of genes encoding mitochondrial proteins are highlighted in bold. *While species overlap suggests a duplication at the root of chordata, the species distribution within the SIRT2 and SIRT3 branches suggest an earlier duplication and multiple losses in many evolutionary branches.

### Tissue-specific expression of novel mitochondrial proteins

**Figure 3:**
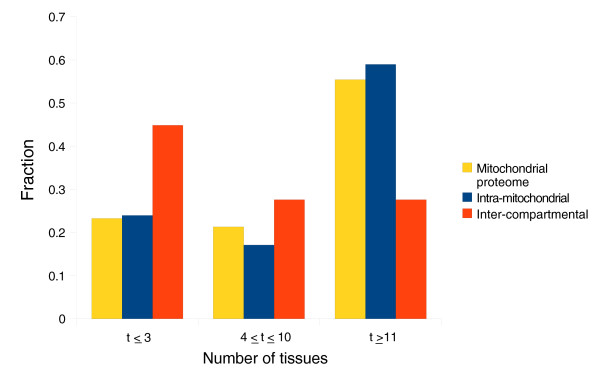
Expression profiles of mitochondrial proteins across a range of mouse tissues. The number of tissues with a detectable protein mass-spectrometry signal (up to 14 tissues investigated in [4]) is shown. The height of the bar represents the fraction of proteins - of intra-mitochondrial or inter-compartmental duplication origins - expressed in a tissue-specific manner (up to three tissues), widely expressed (in more than ten tissues) or expressed in a moderate number of tissues. Figure S2 in Additional data file 1 presents the data in more detail.

Using mass spectrometry total peak intensity data available for 14 different mouse tissues [4], we performed quantitative analysis of tissue-specific protein expression by counting the number of tissues in which the protein was detected (specifically, the number of tissues with log_10 _peak intensities of at least 7). A typical mitochondrial protein is abundantly expressed and detectable in 12 (median value) out of 14 tissues (Table S12 in Additional data file 1). Only proteins that underwent inter-compartmental duplications are expressed in significantly fewer tissues (median 5; *P *< 0.01 using a two-sided Wilcoxon rank sum test performed pairwise with other datasets). These novel mitochondrial proteins (proteins that possess a non-mitochondrial paralog and a non-mitochondrial yeast ortholog) more often exhibit a tissue specific expression pattern with 45% expressed in three tissues or fewer (compared to the mitochondrial average of 23%), and are more rarely widely expressed (in more than 10 tissues; 28% novel mitochondrial proteins compared to 55% on average) (Figure 3).

### Subcellular differentiation via independent gene duplications

**Figure 4:**
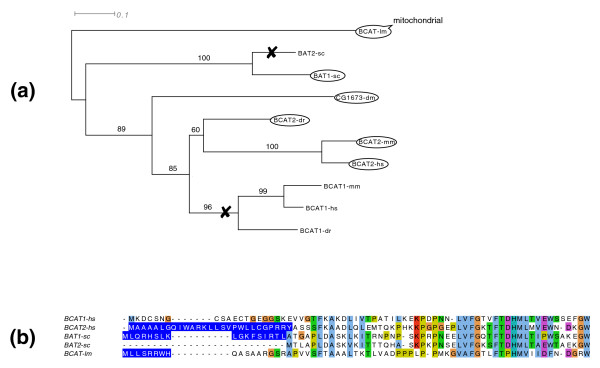
Evolution of mitochondrial localization for the branched-chain-amino-acid aminotransferases family. **(a) **Gene tree generated by PhyML [70] for vertebrates, yeast and outgroup species that speciated before duplication events. Bootstrap values (100 repetitions) are shown on the internal branches. Proteins surrounded by an oval are localized to mitochondria; loss of the mitochondrial localization is marked by a cross. **(b) **Clustal W [71] alignment of the amino-terminal region of orthologs. The predicted targeting sequences are highlighted in blue. Abbreviations: hs. *Homo sapiens*; mm, *Mus musculus*; dm, *Drosophila melanogaster*; dr, *Dano rerio*; lm, *L. major*; sc, *S. cerevisiae*.

While tracing the history of duplications that extend the mitochondrial proteome, one can imagine, in the most drastic scenario, that independent duplications in unrelated lineages with subsequent parallel relocalizations to mitochondria could lead to a convergent evolution in the mitochondrial protein content. Several paralogs present this unusual evolutionary pattern (Table 3). For example, branched-chain-amino-acid aminotransferase underwent duplication at the root of vertebrates, in addition to an independent event in yeast as a result of whole genome duplication. In both species one copy is targeted to the mitochondria (BCAT2 in human), the other is cytosolic (BCAT1). In the case of this gene family, the analysis of distant orthologs for the presence/absence of the targeting signal sheds light on the likely ancestral localization. Using MitoProt II [39] and TargetP [38] the signal can be detected in the fly sequence as well as *Leishmania major *orthologs, suggesting that the ancestral BCAT protein was part of the mitochondrial proteome in the ancestor of human and yeast (Figure 4).

**Table 3:** Independent duplications and parallel relocalizations in the human and yeast lineages have happened multiple times during evolution

	Human		Yeast	
		
Family	Mitochondrial	Non-mitochondrial	Mitochondrial	Non-mitochondrial
Thioredoxins	TXN, TXN2	TXNDC2	TRX3	TRX1, TRX2
Glutaredoxins	GLRX2	GLRX, GLRXL	GRX2	GRX1 (nucleus)
Isocitrate dehydrogenases [NADP]	IDH2	IDH1	IDP1	IDP2, IDP3 (peroxisome)
Branched-chain-amino-acid aminotransferases	BCAT2	BCAT1	BAT1	BAT2
Serine hydroxymethyltransferases	SHMT2	SHMT1	SHM1	SHM2

Rows show proteins targeted to mitochondria and their paralogs targeted to other subcellular compartments in both human and yeast. Non-mitochondrial homologs (both human and yeast) are cytosolic if not indicated otherwise.

### The growth of the mitochondrial proteome by gene duplication

Knowing the homology of proteins with a determined localization in human and yeast, we reconstructed the (partial) protein complement of mitochondria of the common ancestor of human and yeast, comprising circa 200 proteins in total. Starting with this ancestral proteome, we counted 128 duplications of mitochondrial proteins in the human lineage, including intra-mitochondrial duplications and proteins novel to the mitochondria (relocalizations following the duplication of non-mitochondrial proteins). As not all types of evolutionary events allow us to easily infer the ancestral localization, this puts a lower bound on the protein count, concluding that the metazoan mitochondrion in the human lineage expanded by 64% (128 out of 200) by means of gene duplication and relocalization since the evolutionary split with the yeast lineage (see Materials and methods for details). These counts are likely to be an underestimate of a real mitochondrial proteome expansion, as we disregard proteins without recognizable orthologs in *S. cerevisiae *that appeared in the metazoan lineage.

## Discussion and conclusions

Our investigation reveals a dynamic mitochondrial proteome and paints a picture of a eukaryotic organelle with a functional repertoire evolving by gene duplication. In the absence of gene duplication, we find little room for functional diversification of the mitochondrial proteome by relocalization of proteins. The subcellular localization of proteins that did not duplicate since the divergence of human and yeast is almost always conserved in evolution, with a few notable exceptions. In the presence of duplication events the mitochondrion expanded via two major modes. In the first, more conservative mode, intra-mitochondrial duplications expanded the mitochondrial proteome by duplication of proteins that were already localized to mitochondria. In the second and a more radical mode of proteome growth, inter-compartmental duplications expanded the metazoan and human mitochondrial proteome by the duplication of non-mitochondrial proteins and redirecting the newly arisen gene products to the mitochondria.

The two modes of proteome expansion comprise different functional protein classes. Duplications of genes responsible for carbohydrate metabolism, calcium ion binding and various forms of transport appear to be specific to intra-mitochondrial protein duplications, whereas cofactor binding, intramolecular oxidoreductases, ceramide kinase and rRNA methylation functions are more often associated with duplicates that have novel mitochondrial localization.

Intra-mitochondrial duplications that expanded the repertoire of transport proteins are exemplified by two duplications of TIMM8A/B and TIMM17A/B proteins. Expression of both paralogs leads to distinct variants of the intermembrane complexes TIMM8-TIMM13 and Tim23 embedded in the inner membrane [40-42] (Additional data file 1). The Pyruvate dehydrogenase (PDH) complex, which participates in carbohydrate metabolism (a functional class significantly enriched among intra-mitochondrial duplications), underwent intra-mitochondrial duplications at various points in evolution (E1-beta subunit duplicated before the divergence of bilateria; E2 subunit duplicated before the divergence of chordates; E1-alpha subunit duplicated before the divergence of eutheria). The duplication pattern of post-translational regulators of the PDH complex differs from that of the complex itself. The inactivating phosphorylation of the PDH complex is carried out by four paralogs of PDH kinase, and all duplication events occurred before the divergence of the vertebrates. Prior to the catalytic activation, PDH must be dephosphorylated by one of the two paralogous proteins: PDP1 (PPM2C) and PDP2. PDP1, in contrast to its paralog, is activated by calcium ions and, therefore, might mediate the effects of calcium-mobilizing hormones [43]. It is difficult to establish the evolutionary origin of a domain responsible for the binding with Ca^2+^, as the binding site is created upon the formation of a complex with the E2 subunit of the PDH complex and requires the lipoyl groups of E2 [44]. Nevertheless, the calcium-dependence of PDP1 is consistent with a trend present in mitochondrial proteins. We identify duplications of Ca^2+^-binding mitochondrial solute carriers [45], as well as proteins responsible for calcium-sensitive mitochondrial trafficking along microtubules [46,47]. Overall, 11 out of 23 of the calcium ion binding proteins originate from intra-mitochondrial duplications that occurred at the root of vertebrates (*P *< 7e-4, [GO:5509]).

In general, it appears that the regulation of cellular complexes is more evolutionarily recent than the complexes they control. That the duplications of the PDH complex occurred before the vertebral duplications of their regulators, kinases and phosphatases, is not a unique case. Also, the soluble mitochondrial matrix deacetylase SIRT3 has a relatively recent origin, and was shown to augment Complex I activity by binding with the 39 kDa subunit of Complex I, NDUFA9 [48]. It is known that the growth of many mitochondrial protein complexes occurred early in evolution, with mitochondrial Complex I and the mitochondrial ribosome expanding significantly at the root of eukaryotes [49-51]. Interestingly, regulators of activity of the complexes via phosphorylation and dephosphorylation (as for PDH) or deacetylation (Complex I) did not appear concomitantly in evolution and were not adapted from existing regulators, but emerged long after the metazoan diversification.

When analyzing duplications of proteins that expanded the mitochondrial proteome, it would be interesting to know the selective forces driving duplication events. We show that the novel mitochondrial localization that is detectable at the sequence level has been gained rapidly after the duplication event. On the one hand, we know that only a small fraction of duplicated genes is retained in the genome in the long term, and this holds also for large-scale genomic events such as whole genome duplication [52]. On the other hand, the acquisition of an amino-terminal targeting signal coinciding with the gene duplication event could provide the rationale for the retention of the duplicated gene. As the change of localization alters the role of a protein in the cell, it could be accompanied by further functional diversification. This diversification may be extensive, even for relatively recent duplications, as in the case of HTRA2 protease (Table 2). The membrane-bound HTRA2, unlike its secreted paralogs, promotes or induces cell apoptosis through caspase-dependent and -independent pathways [53] and its loss of function mutations cause neurodegeneration and Parkinson's disease [54].

Analysis of the timing of duplication events reveals that the majority of inter-compartmental duplications occurred further back in time than the genomic trend would suggest and that they contributed little to the expansion of the mitochondrial proteome in the vertebrate lineage. The fact that most inter-compartmental duplications occurred before animals diverged suggests that cellular differentiation is partly responsible for inter-compartmental duplications. We propose that the inter-compartmental duplicated proteins could have helped to satisfy the variable energy demands that emerging metazoan tissues presented. There is some anecdotal evidence that could support this hypothesis. For example, the pattern of tissue-specific expression of TOP1MT (Table 2) has adapted to meet the requirements for higher mitochondrial activity in specific organs - for example, skeletal muscle, heart, and brain [55]. Additionally, we observed that inter-compartmental duplications/relocalizations are characterized by a more narrow, tissue-specific expression than average mitochondrial proteins (see Table S12 and Figure S2 in Additional data file 1).

Our quantitative results of the evolution of the mitochondrial proteome match anecdotal evidence for the role of inter-compartmental duplications in the expansion of the proteomes of other eukaryotic organelles. Some pathways and key enzymes were known to have duplicated between plastids and other cellular compartments [56], as observed in the case of sulfate assimilation and cysteine biosynthesis found in the chloroplasts, cytosol and mitochondria of plants [57]. In addition, the evolutionary history of 12 Calvin cycle enzymes shows that plant proteins encoded by the nucleus have relocalized to alternative compartments, regardless of their origin, cyanobacterial or otherwise [58].

With 87% of mitochondrial proteins preserving their ancestral compartment between human and yeast, a gene duplication event appears to be a necessary prerequisite to release the localization constraint, allowing nascent proteins to be retargeted to distinct compartments. We therefore conclude that non-mitochondrial protein duplications followed by the gain of a novel mitochondrial localization comprise a qualitatively and quantitatively important mode of expansion of the mitochondrial proteome.

## Materials and methods

### Mitochondrial proteomes

Mammalian nuclear-encoded mitochondrial proteins were downloaded from MitoCarta, the state-of-the-art compendium of the human mitochondrial proteome established using combination of experimental identification, bioinformatic analysis, and literature curation [4]. We mapped 1,001 human orthologous proteins onto Ensembl identifiers using human-mouse ortholog lists from Ensembl v44 (April 2007) [59] and Mouse Genome Database [60]. For yeast, to assure specificity of its mitochondrial proteome, a reference set was downloaded from the MitoP2 database [61]. This set of 545 proteins contains published experimental data based on various studies [18,22-24] and was subsequently manually curated. To exclude non-confirmed mitochondrial proteins, for which a mitochondrial localization was only predicted or derived from early high-throughput studies, we also required mitochondrial proteins to be present among 851 proteins from the most comprehensive dataset of the yeast mitochondrial proteome to date [19]. The proteomes selected as described assure few false positive proteins, but do not completely cover mitochondrial protein content. Because of the incomplete coverage, the absence of evidence for mitochondrial localization cannot be taken as evidence for the absence of mitochondrial localization. For the non-mitochondrial proteins set, only proteins localized to other eukaryotic subcellular compartments were taken into account. This included proteins explicitly assigned to 24 non-mitochondrial compartments as annotated in GO of human genes (see Table S10 in Additional data file 1 for the full list of the compartments), analogous to the non-mitochondrial reference dataset from [62].

### Gene trees of mitochondrial proteins

To take into account the evolutionary history of every protein, including gene losses and duplications, we performed analysis of individual gene trees reconciled with the species phylogeny, as provided by the Ensembl team [59]. The phylogenomic Ensembl pipeline provides a dataset of gene trees across multiple species, constructed using both dS, dN (substitution rates), nucleotide and protein distance measures [63]. These data, together with the standard species tree, informs the gene tree construction performed by the TreeBeST program [64] (L Heng, AJ Vilella, E Birney, R Durbin, in preparation). First, all protein coding genes are queried using WUBLASTP against the whole protein database. Subsequently, a graph of proteins is constructed, with edges created for best reciprocal hits or when score(P1, P2)/max(score(P1, P1), score(P2, P2)) >0.33. Connected components of the graph are extracted and aligned subsequently with MUSCLE [65]. The back-translated multiple alignment is passed to the tree constructing program, TreeBeST, together with the species tree for the reconciliation and the duplication calls on internal nodes, as the coverage of genomes in the Ensembl database provides topologically based timings in order to label duplication events [63]. All human gene trees with a mitochondrial gene product (mitochondrial proteins in either human or yeast) were downloaded from Ensembl database v44 [59]. When integrating datasets from human and yeast for 50% human genes and 46% yeast proteins, we did not detect homologs in the other species, representing a likely gene loss or gain in one of these lineages.

### Unambiguous one-to-one orthologs between human and yeast

The trees for gene families were separated at the speciation branches into opisthokont orthogroups and the number of paralogs in human and yeast lineages was counted. One-to-one unambiguous orthologs were represented by trees with a single gene in both lineages.

### Gene duplications

For each gene family of *n *genes, we infer *n*-1 duplications, each duplication corresponding to an internal tree node. The dating of the duplication was inferred from the analysis of the tree topology, as annotated by the Ensembl team. We use rooted trees of homologous genes, where branching points are labeled with the inferred time of duplication. For example, a gene tree ((GeneA, GeneB):Euteleostomi,(GeneC, GeneD):Euteleostomi):Chordata yields a single chordate duplication that is followed by two vertebrate duplications. For the inter-compartmental duplication a divergence time of a mitochondrial and a closest non-mitochondrial paralog was inferred from the internal node giving rise to the duplication. To asses the quality of gene duplication calls, we used the duplication consistency score [63]. The score measures the intersection of the number of species post-duplication over the union; one expects that most duplications should have the gene persisting in an equally likely manner in subsequent lineages [63]. All of the three duplication datasets (intra-mitochondrial, inter-compartmental or duplications outside mitochondria) had similar, high consistency scores, with median values of 0.85, 0.86, 0.85, respectively (Figure S1 in Additional data file 1). The datasets tested with two-sided Wilcoxon rank sum test do not exhibit statistically significant differences (*P*-value > 0.65).

### Differential localization

Of the differentially localized one-to-one orthologs, we find 17 proteins localized to mitochondria only in human and 16 of these are either reference mitochondrial proteins known from the literature or were experimentally verified in the Pagliarini *et al*. study [4]. For families with gene duplications and differentially localized human paralogs, localization was predicted computationally for only three mitochondrial proteins, with the remaining proteins validated experimentally in the Pagliarini *et al*. study by either green fluorescent protein marker (4 proteins), proteomics approaches (7 proteins) or being part of a mammalian mitochondrial reference set based on the literature curation (15 proteins).

### *A. thaliana *orthologs

Of the one-to-one human-yeast orthologs, 104 possess an ortholog in plants (determined using the homologene database [66] and 27 were found in mitochondria in Heazlewood *et al*. [20]. With regard to intra-mitochondrial duplications, 47 plant orthologs were found, 23 of which are in the mitochondria.

#### Estimation of the expansion of the mitochondrial proteome

We identified 122 unambiguous one-to-one nuclear encoded gene products with a reliable mitochondrial localization in human and yeast (Table S1 in Additional data file 1), with 17 differentially localized orthologs likely to be mitochondrial gains in the human lineage (see Results). Genes that underwent duplications originated from at least 66 ancestral opisthokont genes (for which we can find at least one protein from the family in mitochondria of both human and yeast; family counts are 53 + 8 + 4 + 1 from Table S4 in Additional data file 1, with each family stemming from a single ancestral gene), or 78 if we add families with uncertain common ancestry (mitochondrial only in human; an additional 12 families). This, together with one-to-one orthologs, gives 188 to 200 ancestral proteins. Given the present human mitochondrial protein compendium, restricted to proteins with an ortholog in yeast with a known localization, we arrive at 128 to 140 mitochondrial acquisitions in the human lineage. Given 188 to 200 ancestral mitochondrial proteins and 128 to 140 gains in the metazoan evolutionary branch, we estimate an expansion of the mitochondrial proteome between 64% (128/200) and 74% (140/188).

### Dating mitochondrial relocalization

For the prediction of the amino-terminal targeting signal in the protein sequences, Target P was used [67] for all known isoforms of a given gene. It is important to mention that the pre-sequence analysis programs do not use homology to known mitochondrial proteins or mitochondria-specific domains as an indicator of presence/absence of targeting signal.

#### Gene Ontology analysis

GO [68] analysis was performed using the BiNGO package [69] using Benjamini and Hochberg false discovery rate correction; corrected *P*-values are specified in Additional data file 1.

## Abbreviations

GO: Gene Ontology; PDH: pyruvate dehydrogenase.

## Authors' contributions

RS and MH conceived the study. RS carried out the analysis and wrote the manuscript. All authors read and approved the final manuscript.

## Additional data files

The following additional data are available with the online version of this paper: supplementary text, Tables S1-S12, and Figures S1 and S2 (Additional data file 1).

## Supplementary Material

Additional data file 1 Supplementary text, Tables S1-S12, and Figures S1 and S2. Click here for file
