# Supplementary material for: Expansion of the human mitochondrial proteome by intra- and inter-compartmental protein duplication
Source: Genome Biol. 2009 Nov 24;10(11):R135. doi: 10.1186/gb-2009-10-11-r135 (PMC3091328; doi:10.1186/gb-2009-10-11-r135)

*Intra-mitochondrial expansion of the protein transport machinery.* TIMM8A (DDP1), subunits of the small intermembrane TIMM8-TIMM13 complex, forms a heterohexamer, composed of 3 copies of TIMM8A and 3 copies of TIMM13. TIMM8B, its paralog, likely forms a similar heteromer, as both TIMM8A and TIMM8B were purified in a complex with TIMM13 in independent purifications, but the two paralogs have not been captured in a single protein complex together (Ewing et al. 2007), and the lack of interaction in-vivo is supported by tissue specific expression pattern (see below). A similar pattern can be observed for TIMM17, a core subunit of the translocation channel of the inner membrane TIM23 complex (Bömer et al. 1996). In vertebrates, two paralogs encode the proteins TIMM17A and TIMM17B subunits of the inner membrane Tim23 complex. The duplication of the TIMM17 gene led to two distinct versions of the complex, one containing hTim17a and one containing hTim17b (Bauer et al. 1999). Although the interaction patterns and gene expression patterns of TIMM8 and TIM17 genes suggest functional differentiation, it remains to be determined whether the protein complex isoforms reflect the development of different substrate specificities during the evolution of mammals.

*Relocalization dataset*. With 87% of single-copy proteins preserving their ancestral compartment, a gene duplication event appears to be a necessary prerequisite to release the localization constraint and allow nascent proteins to be retargeted to distinct compartments. But is the relocalization a genuine effect or a result of incorrectly specified localization? To avoid this difficulty, we used the most recent compendium of human mitochondrial proteome with estimated false discovery rate of 10% (Pagliarini et al., 2008). Closer inspection of these differentially targeted paralogs (potentially more prone to errors) reveals that the mitochondrial location has been predicted computationally for only three proteins from this set, and that all the remaining cases are supported by experimental evidence (see Methods). We also observe significant functional coherence among inter-compartmental duplications, not expected if the proteins' locations were erroneous. Moreover, the computational analysis reveals the presence of targeting signal in ~50% mitochondrial proteins of this type (5x more often than in their non-mitochondrial paralogs), consistent with our knowledge about mitochondrial proteins (Sickmann et al., 2003, Bolender et al., 2008). We therefore conclude that a significant fraction of the metazoan mitochondrial proteome comes from ancestral non-mitochondrial proteins: a duplication accompanied by gain of mitochondrial localization signal is a major mode of expansion of mammalian mitochondrion, expanding the repertoire of catalytic activity, ion transport and rRNA modification.

***Table S1.*** 1-1 human-yeast orthologs, where at least one ortholog is a member of reference mitochondrial dataset. Column descriptions: human mito - human mitochondrial protein, from a reference dataset (see Text), yeast mito - yeast mitochondrial protein, hugo- name of the human protein, mitocarta - type of experimental support for the mitochondrial localization (Pagliarini et al., 2008), yeast gene name - name of the yeast mitochondrial ortholog, arabidopsis mito - presence of the protein in plant mitochondria (Heazlewood et al., 2004).

| human mito | yeast mito | hugo | yeast gene name | mitocarta | **arabidopsis mito** |
| --- | --- | --- | --- | --- | --- |
| 1 | 1 | CECR5 | YKR070W | proteomics | 0 |
| 1 | 1 | SLC25A1 | CTP1 | known | 0 |
| 1 | 1 | SOD1 | SOD1 | known | 0 |
| 1 | 1 | ATP5O | ATP5 | strong_computational_pred | 1 |
| 1 | 1 | OXA1L | OXA1 | known | 0 |
| 1 | 1 | ATP5A1 | ATP1 | known | 0 |
| 1 | 1 | HSCB | JAC1 | gfp_validated | 0 |
| 1 | 1 | FECH | HEM15 | known | 0 |
| 1 | 1 | PISD | PSD1 | known | 0 |
| 1 | 1 | ACO2 | ACO1 | known | 0 |
| 1 | 1 | GFER | ERV1 | gfp_validated | 0 |
| 1 | 1 | SAMM50 | SAM50 | known | 0 |
| 1 | 1 | COQ6 | COQ6 | known | 0 |
| 1 | 1 | ISCA2 | ISA2 | proteomics | 0 |
| 1 | 1 | DLST | KGD2 | known | 1 |
| 1 | 1 | POLRMT | RPO41 | known | 0 |
| 1 | 1 | ENSG00000183596 | PAM16 | known | 0 |
| 1 | 1 | DNAJA3 | MDJ1 | known | 0 |
| 1 | 1 | TRNT1 | CCA1 | known | 0 |
| 1 | 1 | ATP5D | ATP16 | known | 1 |
| 1 | 1 | LIAS | LIP5 | known | 0 |
| 1 | 1 | TIMM22 | TIM22 | known | 0 |
| 1 | 1 | MIPEP | OCT1 | known | 0 |
| 1 | 1 | TIMM13 | TIM13 | known | 0 |
| 1 | 1 | MTFMT | FMT1 | known | 0 |
| 1 | 1 | CLPX | MCX1 | known | 0 |
| 1 | 1 | COQ7 | CAT5 | known | 0 |
| 1 | 1 | GLRX5 | GRX5 | proteomics | 0 |
| 1 | 1 | CHCHD4 | MIA40 | known | 0 |
| 1 | 1 | EARS2 | MSE1 | gfp_validated | 0 |
| 1 | 1 | NDUFAB1 | ACP1 | known | 1 |
| 1 | 1 | MRPL15 | MRPL10 | known | 0 |
| 1 | 1 | OXSM | CEM1 | known | 0 |
| 1 | 1 | COX5A | COX6 | known | 0 |
| 1 | 1 | TUFM | TUF1 | known | 1 |
| 1 | 1 | TIMM44 | TIM44 | known | 0 |
| 1 | 1 | IDH3A | IDH2 | proteomics | 1 |
| 1 | 1 | YARS2 | MSY1 | known | 0 |
| 1 | 1 | COQ2 | COQ2 | strong_computational_pred | 0 |
| 1 | 1 | ACN9 | ACN9 | proteomics | 0 |
| 1 | 1 | MRPL46 | MRPL17 | known | 0 |
| 1 | 1 | POLG | MIP1 | known | 0 |
| 1 | 1 | UQCRB | QCR7 | strong_computational_pred | 0 |
| 1 | 1 | LARS2 | NAM2 | known | 0 |
| 1 | 1 | MRPL17 | MRPL8 | known | 0 |
| 1 | 1 | COX10 | COX10 | known | 0 |
| 1 | 1 | COQ9 | COQ9 | proteomics | 1 |
| 1 | 1 | ATPAF2 | ATP12 | known | 0 |
| 1 | 1 | SLC25A20 | CRC1 | known | 1 |
| 1 | 1 | MRPL13 | MRPL23 | known | 0 |
| 1 | 1 | DLD | LPD1 | known | 1 |
| 1 | 1 | GTPBP3 | MSS1 | known | 0 |
| 1 | 1 | SLC25A42 | YPR011C | proteomics | 0 |
| 1 | 1 | CYC1 | CYT1 | known | 1 |
| 1 | 1 | PET112L | PET112 | known | 0 |
| 1 | 1 | ATP5B | ATP2 | known | 1 |
| 1 | 1 | LDHD | DLD1 | known | 0 |
| 1 | 1 | ETFDH | YOR356W | known | 0 |
| 1 | 1 | SLC25A26 | PET8 | known | 0 |
| 1 | 1 | TIMM10 | MRS11 | known | 0 |
| 1 | 1 | GLDC | GCV2 | known | 1 |
| 1 | 1 | MRPS12 | YNR036C | known | 0 |
| 1 | 1 | C11orf79 | EMI5 | gfp_validated | 0 |
| 1 | 1 | TIMM50 | TIM50 | known | 0 |
| 1 | 1 | ATP5C1 | ATP3 | known | 1 |
| 1 | 1 | CRLS1 | CRD1 | known | 0 |
| 1 | 1 | NAGS | ARG5,6 | known | 0 |
| 1 | 1 | NFU1 | NFU1 | proteomics | 0 |
| 1 | 1 | MRPL3 | MRPL9 | known | 1 |
| 1 | 1 | PPTC7 | PTC7 | proteomics | 0 |
| 1 | 1 | PHB | PHB1 | known | 1 |
| 1 | 1 | MRPL27 | MRP7 | computational_pred | 0 |
| 1 | 1 | MRPL11 | MRPL19 | known | 0 |
| 1 | 1 | COX11 | COX11 | proteomics | 0 |
| 1 | 1 | COQ5 | COQ5 | proteomics | 1 |
| 1 | 1 | MRPS5 | MRPS5 | known | 0 |
| 1 | 1 | COX5B | COX4 | known | 0 |
| 1 | 1 | GFM1 | MEF1 | known | 1 |
| 1 | 1 | ENSG00000175536 | LIP2 | proteomics | 0 |
| 1 | 1 | MRPS9 | MRPS9 | known | 0 |
| 1 | 1 | YME1L1 | YME1 | known | 0 |
| 1 | 1 | ETFB | YGR207C | known | 1 |
| 1 | 1 | NARS2 | SLM5 | gfp_validated | 0 |
| 1 | 1 | FDXR | ARH1 | known | 0 |
| 1 | 1 | FXN | YFH1 | known | 0 |
| 1 | 1 | UQCC | CBP3 | known | 0 |
| 1 | 1 | REXO2 | REX2 | proteomics | 0 |
| 1 | 1 | MRPL12 | MNP1 | known | 0 |
| 1 | 1 | SLC25A10 | DIC1 | known | 0 |
| 1 | 1 | GPD2 | GUT2 | known | 1 |
| 1 | 1 | SLC25A16 | LEU5 | known | 0 |
| 1 | 1 | SUPV3L1 | SUV3 | known | 0 |
| 1 | 1 | MARS2 | MSM1 | proteomics | 1 |
| 1 | 1 | BCS1L | BCS1 | known | 0 |
| 1 | 1 | ATAD1 | MSP1 | proteomics | 0 |
| 1 | 1 | MRPL44 | MRPL3 | known | 0 |
| 1 | 1 | COX15 | COX15 | known | 0 |
| 1 | 1 | MRPL43 | MRPL51 | known | 0 |
| 1 | 1 | SURF1 | SHY1 | known | 1 |
| 1 | 1 | SDHB | SDH2 | known | 1 |
| 1 | 1 | MRPS2 | MRP4 | known | 0 |
| 1 | 1 | ALDH4A1 | PUT2 | known | 0 |
| 1 | 1 | PMPCA | MAS2 | known | 0 |
| 1 | 1 | MTG1 | MTG1 | proteomics | 0 |
| 1 | 1 | COQ3 | COQ3 | proteomics | 0 |
| 1 | 1 | RTN4IP1 | YIM1 | known | 0 |
| 1 | 1 | QRSL1 | YMR293C | gfp_validated | 0 |
| 1 | 1 | MECR | ETR1 | known | 1 |
| 1 | 1 | AK2 | ADK1 | known | 1 |
| 1 | 1 | MRPS15 | MRPS28 | known | 0 |
| 1 | 1 | TRIT1 | MOD5 | strong_computational_pred | 0 |
| 1 | 1 | ATPAF1 | ATP11 | known | 0 |
| 1 | 1 | SOD2 | SOD2 | known | 1 |
| 1 | 1 | CPT2 | YAT1 | known | 0 |
| 1 | 1 | ATP5F1 | ATP4 | known | 0 |
| 1 | 1 | WARS2 | MSW1 | known | 0 |
| 1 | 1 | PPOX | HEM14 | known | 0 |
| 1 | 1 | PRDX6 | PRX1 | computational_pred | 0 |
| 1 | 1 | DARS2 | MSD1 | strong_computational_pred | 0 |
| 1 | 1 | IARS2 | ISM1 | proteomics | 0 |
| 1 | 1 | FH | FUM1 | proteomics | 1 |
| 1 | 1 | MT-ATP6 | ATP6 | known | 0 |
| 1 | 1 | MT-CO3 | COX3 | known | 0 |
| 1 | 1 | MT-CO2 | COX2 | known | 0 |
| 1 | 0 | ZADH2 | ZTA1 | proteomics | 1 |
| 1 | 0 | MSRA | MXR1 | proteomics | 0 |
| 1 | 0 | DUT | DUT1 | known | 0 |
| 1 | 0 | ACAA1 | POT1 | strong_computational_pred | 0 |
| 1 | 0 | RNASEH1 | RNH1 | known | 0 |
| 1 | 0 | DUS2L | SMM1 | gfp_validated | 0 |
| 1 | 0 | PLA2G15 | LRO1 | known | 0 |
| 1 | 0 | ABCF2 | ARB1 | known | 0 |
| 1 | 0 | PRDX5 | AHP1 | proteomics | 0 |
| 1 | 0 | DCAKD | YDR196C | gfp_validated | 0 |
| 1 | 0 | THG1L | THG1 | known | 0 |
| 1 | 0 | OTC | ARG3 | known | 0 |
| 1 | 0 | CCT7 | CCT7 | gfp_validated | 0 |
| 1 | 0 | CLPB | HSP104 | proteomics | 0 |
| 1 | 0 | KYNU | BNA5 | proteomics | 0 |
| 1 | 0 | ALDH18A1 | PRO2 | known | 0 |
| 1 | 0 | OAT | CAR2 | known | 0 |
| 0 | 1 | TSEN2 | SEN2 |  |  |
| 0 | 1 | DNM1L | DNM1 |  | 0 |

***Table S2.*** Many of relocalized 1-1 orthologs are found in mutliple compartments. Of 17 proteins found in mitochondria of human, but not yeast, published small-scale experiments indicate multiple localization compartments in at least 8 proteins.

| **gene** | **localization (reference)** |
| --- | --- |
| MSRA | cytosoli+mitochondria (Vougier 2003) |
| DUT | nucleus+mitochondria (Ladner et al., 1996) |
| RNASEH1 | nucleus+mitochondria (Cerritelli ’03) |
| DUS2L | ER+cytosol (Kato 2005) |
| ABCF2 | cytosol+mitochondria (Nougayrède, 2007) |
| PRDX5 | cytosol+peroxisome+mitochondria (Knoops, 1999) |
| THG1L | cytosol+mitochondria (Guo et al., 2004; Mehrle et al., 2006) |
| KYNU | cytosol+mitochondria (Inada 1984) |

***Table S3.*** Intra-mitochondrial duplications. Ancestral gene encoding mitochondrial protein underwent a duplication with all the paralogs targeted to mitochondria. Proteins listed in a single row are homologs.

| **yeast genes** | **human genes** |
| --- | --- |
| PDB1 | PDHB, BCKDHB |
| CYC1, CYC7 | CYCS |
| PKP1 | PDK2, PDK4, PDK1, PDK3 |
| SDH1, YJL045W | SDHA |
| MSR1 | RARS2, RARS |
| ALD4, ALD5, ALD6 | ALDH2, ALDH1B1 |
| GLO4 | HAGH, PNKD, ETHE1 |
| PET9, AAC3, AAC1 | SLC25A5, SLC25A6, SLC25A4, SLC25A31 |
| FSF1 | SFXN3, SFXN1, SFXN2, SFXN5 |
| ODC1, ODC2 | SLC25A21 |
| YMC2, YMC1 | SLC25A29, C14orf68, ENSP00000274513 |
| AGC1 | SLC25A12, SLC25A13 |
| OAC1 | SLC25A34, SLC25A35 |
| TOM70, TOM71 | TOMM70A |
| TOM40 | TOMM40, TOMM40L |
| LSC2 | SUCLA2, SUCLG2 |
| ABC1 | ADCK4, CABC1 |
| SHM1 | SHMT1, SHMT2 |
| IDH1 | IDH3G, IDH3B |
| GEM1 | RHOT2, RHOT1 |
| MRF1 | MTRF1L, MTRF1 |
| ARG8 | AGXT2L2, AGXT2 |
| MRS4 | SLC25A37, SLC25A28 |
| RIM2 | SLC25A36, SLC25A33 |
| CYC3, CYT2 | HCCS |
| SAL1 | SLC25A23, SLC25A24, SLC25A25 |
| TIM8 | TIMM8A, TIMM8B |
| GCV1 | SARDH, ENSP00000288050, DMGDH, AMT |
| HEM1 | ALAS1, ALAS2, GCAT |
| KGD1 | OGDHL, OGDH, DHTKD1 |
| POR1, POR2 | VDAC2, VDAC1, VDAC3 |
| PTC5 | ENSP00000309548, PPM2C |
| ADK2 | AK3, ENSP00000370456 |
| SYM1 | ENSP00000247712, PXMP2, MPV17 |
| COQ1 | PDSS1, PDSS2 |
| LAT1 | DLAT, PDHX |
| SSC1, ECM10, SSQ1 | HSPA9 |
| FMP32 | CCDC90B, CCDC90A |
| TIM17 | TIMM17A, TIMM17B |
| CAT2 | CROT, CPT1A, CPT1B, CPT1C, CRAT, CHAT |
| COX13 | COX6A1, COX6A2 |
| MGE1 | GRPEL2, GRPEL1 |
| NUC1 | ENDOG, ENDOGL1 |
| PDA1 | PDHA1, PDHA2 |
| MAS1 | PMPCB, UQCRC1 |
| TIM9 | TIMM9, FXC1 |
| HFA1 | ACACA, ACACB |
| SCO1, SCO2 | SCO1 |
| OLI1 | ATP5G3, ATP5G2, ATP5G1 |
| MAE1 | ME1, ME3, ME2 |
| LSC1 | SUCLG1, ACLY |
| PIC2, MIR1 | SLC25A3 |

***Table S4.*** Detailed gene counts for all possible topologies of gene duplications with orthologs in human and yeast. The first four columns describe topology of the orthogroup: presence (black) or absence (white) of human genes encoding mitochondrial paralogs, presence/absence of human non-mitochondrial paralogs, and respectively for yeast in the the columns 3 and 4. Please note that the first row indicates intra-mitochondrial duplications and the second inter-compartmental gene duplication (human paralog relocalized to mitochondria). Remaining columns describe number of gene families with the given topology (orthogroups, column 5) and specific gene counts (columns 6-9).

| **human mito** | **human non-mito** | **yeast mito** | **yeast non-mito** | **orthogroups** | **human mito gene count** | **human non-mito gene count** | **yeast mito gene count** | **yeast non-mito gene count** |
| --- | --- | --- | --- | --- | --- | --- | --- | --- |
|  |  |  |  | 53 | 118 | 0 | 69 | 0 |
|  |  |  |  | 26 | 29 | 72 | 0 | 29 |
|  |  |  |  | 12 | 22 | 0 | 0 | 22 |
|  |  |  |  | 8 | 14 | 0 | 8 | 8 |
|  |  |  |  | 4 | 4 | 4 | 4 | 0 |
|  |  |  |  | 1 | 2 | 1 | 1 | 2 |
|  |  |  |  | 0 | 0 | 0 | 0 | 0 |
|  |  |  |  | 0 | 0 | 0 | 0 | 0 |

***Table S5.*** GO categories enriched among intra-mitochondrial duplications, on the background of mitochondrial proteome (Benjamini & Hochberg False Discovery Rate corrected p-value < 1e-3). Figure 1 from the manuscript depicts non-redundant categories listed in this table, while omitting GO cellular compartment classification.

| **GO-ID** | **corr p-value** | **Description** |
| --- | --- | --- |
| 44429 | 5.23E-007 | mitochondrial part |
| 44422 | 1.60E-005 | organelle part |
| 44446 | 1.60E-005 | intracellular organelle part |
| 31966 | 6.50E-005 | mitochondrial membrane |
| 5975 | 1.49E-004 | carbohydrate metabolic process |
| 5740 | 1.49E-004 | mitochondrial envelope |
| 16624 | 1.91E-004 | oxidoreductase activity, acting on the aldehyde or oxo group of donors, disulfide as acceptor |
| 31975 | 1.91E-004 | envelope |
| 31967 | 1.91E-004 | organelle envelope |
| 31090 | 2.03E-004 | organelle membrane |
| 6810 | 5.77E-004 | transport |
| 16020 | 5.77E-004 | membrane |
| 51234 | 6.02E-004 | establishment of localization |
| 5743 | 6.20E-004 | mitochondrial inner membrane |
| 16021 | 6.20E-004 | integral to membrane |
| 31224 | 6.65E-004 | intrinsic to membrane |
| 19866 | 6.84E-004 | organelle inner membrane |
| 5509 | 6.92E-004 | calcium ion binding |
| 5478 | 8.20E-004 | transporter activity |
| 6006 | 9.98E-004 | glucose metabolic process |

*Table S6.* Human proteins duplicated and relocalized with one of the copies found in mitochondria, other copy targeted to other compartment.

| **mitochondrial** | **non-mitochondrial** |
| --- | --- |
| SEPT4 | SEPT2, SEPT5, SEPT7 |
| VAMP1, VAMP8 | VAMP5, VAMP2, VAMP7, VAMP4 |
| ARMC4 | CTNNB1, JUP |
| SLC22A4 | SVOP, SLC22A5, SLC22A12, SLC22A11, SLC22A6, SLC22A7, SLC22A13, SLC22A14, SLC22A1, SLC22A2, SLC22A3, SV2C, SV2A, SV2B |
| HTRA2 | IGFBP7, IGFBPL1, KAZALD1, HTRA1, HTRA3 |
| L2HGDH | RASGRF1, RASGRP3, RASGRP2, RASGRP4 |
| SLC16A1 | SLC16A6, SLC16A3, SLC16A8, SLC16A7, SLC16A5, SLC16A4, SLC16A2 |
| SIRT3 | SIRT2 |
| TOP1MT | TOP1 |
| PDE12 | CNOT6, CNOT6L |
| TCIRG1 | ATP6V0A2, ATP6V0A4 |
| SECISBP2 | COPS2, PSMD11 |
| HSD3B1 | HSD3B7, NSDHL |
| NUDT13 | NUDT12 |
| CYB5B | FADS2, FADS1 |
| HSDL1 | HSD17B3, HSD17B12 |
| APEX2 | APEX1 |
| TFB2M, TFB1M | DIMT1L |
| NUDT8 | NUDT7 |
| PECI | ENSG00000182415, CDY2B, CDY1, ENSG00000172288, CDYL, CDYL2 |
| HINT2 | HINT1 |
| GOT2 | GOT1 |
| NFXL1 | NFX1 |
| CERK, AGK | CERKL, SPHK1, SPHK2 |
| ACOX3 | ACOXL, ACOX2 |
| SPTLC2 | SPTLC3 |

***Table S7.*** GO categories enriched among inter-compartmental duplications (Benjamini & Hochberg False Discovery Rate corrected p-values).

| **GO-ID** | **corr p-value** | **Description** |
| --- | --- | --- |
| 5739 | 3.04E-014 | mitochondrion |
| 44444 | 4.97E-007 | cytoplasmic part |
| 44429 | 1.91E-006 | mitochondrial part |
| 31966 | 2.40E-005 | mitochondrial membrane |
| 5740 | 2.59E-005 | mitochondrial envelope |
| 43231 | 1.07E-004 | intracellular membrane-bounded organelle |
| 43227 | 1.07E-004 | membrane-bounded organelle |
| 5737 | 1.07E-004 | cytoplasm |
| 31967 | 1.54E-004 | organelle envelope |
| 31975 | 1.54E-004 | envelope |
| 1729 | 3.45E-004 | ceramide kinase activity |
| 8649 | 3.45E-004 | rRNA methyltransferase activity |
| 16433 | 3.45E-004 | rRNA (adenine) methyltransferase activity |
| 179 | 3.45E-004 | rRNA (adenine-N6,N6-)-dimethyltransferase activity |
| 43229 | 1.11E-003 | intracellular organelle |
| 43226 | 1.11E-003 | organelle |
| 48037 | 1.25E-003 | cofactor binding |
| 5624 | 1.28E-003 | membrane fraction |
| 5626 | 1.36E-003 | insoluble fraction |
| 3824 | 1.40E-003 | catalytic activity |
| 154 | 3.27E-003 | rRNA modification |
| 16863 | 4.30E-003 | intramolecular oxidoreductase activity, transposing C=C bonds |
| 5743 | 4.36E-003 | mitochondrial inner membrane |
| 267 | 4.53E-003 | cell fraction |
| 44424 | 4.53E-003 | intracellular part |
| 19866 | 4.53E-003 | organelle inner membrane |
| 31971 | 4.53E-003 | mitochondrial intermembrane space |
| 31970 | 5.24E-003 | organelle envelope lumen |
| 4143 | 5.96E-003 | diacylglycerol kinase activity |
| 31090 | 6.52E-003 | organelle membrane |
| 8173 | 8.59E-003 | RNA methyltransferase activity |

***Table S8.*** Yeast protein localization for orthologs of inter-compartmental duplications in human.

| **yeast gene** | **cell compartment** |
| --- | --- |
| CDC11 | cytoskeleton |
| SNC2 | cytoplasmic vesicle,endosome,Golgi apparatus |
| SNC1 | cytoplasmic vesicle,endosome,Golgi apparatus |
| VAC8 | vacuole,cytoskeleton |
| PHO84 | plasma membrane |
| NMA111 | nucleus |
| CDC25 | plasma membrane |
| MCH5 | plasma membrane |
| MCH4 | vacuole |
| HST2 | nucleus |
| TOP1 | nucleus |
| CCR4 | nucleus |
| STV1 | vacuole,endosome,Golgi apparatus |
| RPN6 | cytosol |
| ERG26 | endoplasmic reticulum |
| NPY1 | microbody |
| CYB5 | microsome,endoplasmic reticulum |
| IFA38 | endoplasmic reticulum |
| APN2 | nucleus |
| DIM1 | nucleus |
| PCD1 | microbody |
| ECI1 | microbody |
| DCI1 | microbody |
| HNT1 | nucleus |
| AAT2 | microbody |
| FAP1 | nucleus |
| LCB4 | plasma membrane,endoplasmic reticulum,Golgi apparatus |
| POX1 | microbody |
| LCB2 | microsome,endoplasmic reticulum |

***Table S9.*** Timing of duplications of proteins with respect to their type. Intra-mitochondrial, inter-compartmental and duplications of proteins located to other compartments are indicated in columns.

| **timing** | **intra-mitochondrial** | **inter-compartmental** | **outside-mitochondria** |
| --- | --- | --- | --- |
| Catarrhini | 0 | 0 | 5 |
| Simiiformes | 0 | 0 | 0 |
| Haplorrhini | 0 | 0 | 0 |
| Primates | 0 | 0 | 0 |
| Euarchontoglires | 0 | 0 | 2 |
| Eutheria | 1 | 0 | 58 |
| Theria | 1 | 0 | 9 |
| Mammalia | 0 | 0 | 14 |
| Amniota | 1 | 1 | 8 |
| Tetrapoda | 1 | 0 | 8 |
| Sarcopterygii | 0 | 0 | 0 |
| Euteleostomi | 32 | 8 | 238 |
| Teleostomi | 0 | 0 | 0 |
| Gnathostomata | 0 | 0 | 0 |
| Vertebrata | 0 | 0 | 0 |
| Craniata | 0 | 0 | 0 |
| Chordata | 4 | 1 | 30 |
| Deuterostomia | 0 | 0 | 0 |
| Coelomata | 4 | 2 | 33 |
| Bilateria | 21 | 17 | 136 |

***Table S10.*** The list of GO terms (Calvo et al., 2007, Pagliarini et al., 2008) corresponding to 24 non-mitochondrial eukaryotic subcellular compartments. Proteins also localized to mitochondria according to GO terms were excluded from this dataset ultimately yielding ~9000 human and ~3000 non-mitochondrial gene products.

| cell surface | GO:0009986 |
| --- | --- |
| chromatin | GO:0000785 |
| cilium | GO:0005929 |
| coated pit | GO:0005905 |
| cytoplasmic vesicle | GO:0016023 |
| cytoskeleton | GO:0005856 |
| cytosol | GO:0005829 |
| cytosolic ribosome | GO:0005830 |
| endoplasmic reticulum | GO:0005783 |
| endosome | GO:0005768 |
| extracellular matrix | GO:0031012 |
| extracellular region | GO:0005576 |
| flagellum | GO:0009434 |
| Golgi apparatus | GO:0005794 |
| intercellular junction | GO:0005911 |
| melanosome | GO:0042470 |
| microbody | GO:0042579 |
| microsome | GO:0005792 |
| nucleus | GO:0005634 |
| plasma membrane | GO:0005886 |
| thylakoid | GO:0009579 |
| tight junction | GO:0005923 |
| ubiquitin ligase complex | GO:0000151 |
| vacuole | GO:0005773 |

***Table S11***. After the divergence of vertebrates, 16 duplications of genes encoding mitochondrial proteins took place in the primate lineage.

| **Mitochondrial protein** | **Primate paralog(s)** |
| --- | --- |
| SERHL2 | SERHL |
| ACOT2 | ACOT1 |
| GLRX | GLRXL |
| CKMT1A | CKMT1B |
| MCART1 | MCART2 |
| ATAD3C | ATAD3B |
| CHCHD2 | CHCHD9 |
| DHRS4 | DHRS4L2 |
| ACSM2A | ACSM2B |
| TMEM14C | TMEM14B |
| HSD3B1 | HSD3B2 |
| HIGD2A | HIGD2BP |
| FAHD2A | FAHD2B |
| TIMM23 | TIMM23B |
| CYP11B2 | CYP11B1 |
| GLUD1 | GLUD2 |

*Table S12.* Median and average number of tissues with a protein expressed. “no-duplication" dataset is comprised of 1-1 human-yeast orthologous proteins with mitochondrial localization in both species.

| **protein dataset** | **median number of tissues** | **average number of tissues** |
| --- | --- | --- |
| all mitochondrial proteins (mitocarta) | 12 | 9.08 |
| no duplication (1-1 orthologs) | 13 | 10.48 |
| intra-mitochondrial duplications | 12 | 9.23 |
| inter-compartmental duplications | 5 | 6.13 |

***Figure S1****. Duplication consistency score for different types of duplication. The score measures the intersection of the number of species post-duplication over the union. For the three duplication types we see a similar distribution of scores (p-value>0.65, Wilcoxon rank sum test).*


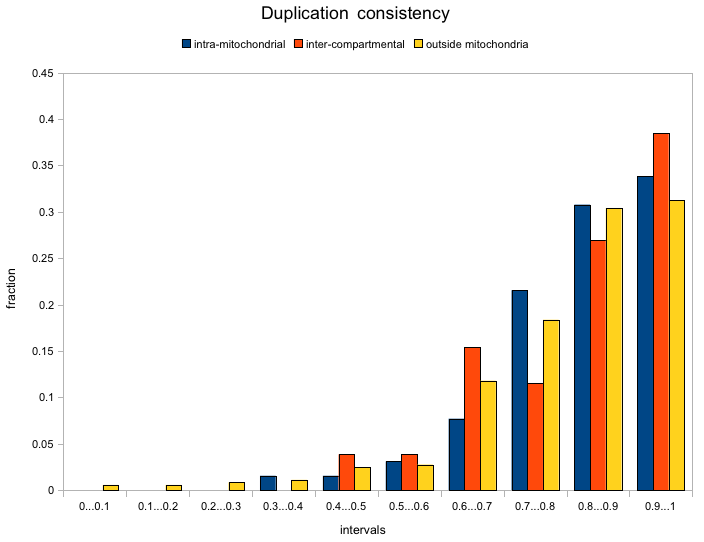


***Figure S2****. Tissue-specific expression of mitochondrial proteins. Number of tissues with detectable protein MS peak (up to 14 tissues investigated in Pagliarini et al., 2008) is plotted against the fraction of proteins in a dataset. “no duplication" dataset is comprised of 1-1 human-yeast orthologous proteins with mitochondrial localization in both species. Only inter-compartmental duplications (expressed with log_10_ peak intensities of at least 7 in median 5 tissues) appear to have significantly narrower expression when tested pairwise with other datasets using a two-sided Wilcoxon rank sum test (P<0.01). Limited expression of inter-compartmental duplications remained significant when we excluded proteins not detected at the required MS peak intensity in any tissue (P<0.02).*


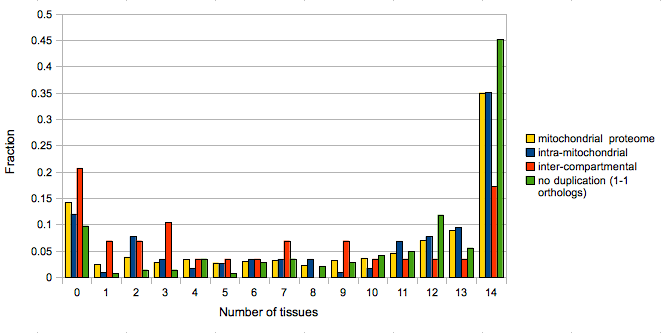

Supplement: Additional data file 1 — Supplementary text, Tables S1-S12, and Figures S1 and S2. [file gb-2009-10-11-r135-S1.DOC]
